# P05 - Wider neck circumference may be related with symptom persistence in children with allergic rhinitis

**DOI:** 10.1186/2045-7022-4-S1-P60

**Published:** 2014-02-28

**Authors:** Süleyman Tolga Yavuz, Bülent Hacıhamdioğlu, Mutluay Arslan

**Affiliations:** 1 Department of Pediatric Allergy, Gulhane Military School of Medicine, Ankara, Turkey; 2 Department of Pediatric Endocrinology, Gulhane Military School of Medicine, Ankara, Turkey; 3 Department of Pediatrics, Gulhane Military School of Medicine, Ankara, Turkey

## Background

Obesity is an established risk factor for the occurrence and severity of atopic diseases, particularly asthma in adults and children. Because asthma and allergic rhinitis share common allergic inflammatory pathways, the aim of the study is to investigate the association between fat distributions that is determined by anthropometric measures including neck circumference and the severity and persistence patterns of children with allergic rhinitis (AR).

## Method

Children with AR who were followed in outpatient department of our unit were consecutively recruited. The diagnosis and classification of AR were made according to GINA guidelines. Anthropometric measures including height, weight, neck circumference (NC), waist circumference and hip circumference were obtained.

## Results

A total of 86 children (63 male, 73.3%) with a mean age of 9.8 ± 2.2 years were included. Mild intermittent rhinitis was diagnosed in 25 (29.1%) patients, mild persistent rhinitis in 13 (15.1%) moderate/severe intermittent rhinitis in 17 (19.8%) and moderate/severe persistent rhinitis in 31 (36.0%) patients. There were no significant differences between children with mild and moderate/severe rhinitis in terms of age, gender and anthropometric measures. However, NC of children with persistent rhinitis were significantly wider than children with intermittent rhinitis (29.8 cm ± 2.7 vs. 28.4 ± 2.1, p=0.013).

## Conclusion

Neck circumference, which is a simple tool of anthropometric measures, is more associated with disease persistence pattern in children with AR when compared with conventional methods.

